# Heart Failure with Preserved Ejection Fraction: The Pathophysiological Mechanisms behind the Clinical Phenotypes and the Therapeutic Approach

**DOI:** 10.3390/ijms25020794

**Published:** 2024-01-08

**Authors:** Laurențiu Stoicescu, Dana Crişan, Claudiu Morgovan, Lucreţia Avram, Steliana Ghibu

**Affiliations:** 1 Internal Medicine Department, Faculty of Medicine, “Iuliu Haţieganu” University of Medicine and Pharmacy, 400000 Cluj-Napoca, Romania; laurentiu.stoicescu@umfcluj.ro (L.S.); or crisan.dana@umfcluj.ro (D.C.); or avram.lucretia@umfcluj.ro (L.A.); 2 Cardiology Department, Clinical Municipal Hospital, 400139 Cluj-Napoca, Romania; 3 Internal Medicine Department, Clinical Municipal Hospital, 400139 Cluj-Napoca, Romania; 4 Preclinical Department, Faculty of Medicine, “Lucian Blaga” University of Sibiu, 550169 Sibiu, Romania; 5 Department of Pharmacology, Physiology and Pathophysiology, Faculty of Pharmacy, “Iuliu Haţieganu” University of Medicine and Pharmacy, 400349 Cluj-Napoca, Romania; steliana.ghibu@umfcluj.ro

**Keywords:** heart failure, HFpEF, pathophysiological mechanism, phenotypes, treatment

## Abstract

Heart failure (HF) with preserved ejection fraction (HFpEF) is an increasingly frequent form and is estimated to be the dominant form of HF. On the other hand, HFpEF is a syndrome with systemic involvement, and it is characterized by multiple cardiac and extracardiac pathophysiological alterations. The increasing prevalence is currently reaching epidemic levels, thereby making HFpEF one of the greatest challenges facing cardiovascular medicine today. Compared to HF with reduced ejection fraction (HFrEF), the medical attitude in the case of HFpEF was a relaxed one towards the disease, despite the fact that it is much more complex, with many problems related to the identification of physiopathogenetic mechanisms and optimal methods of treatment. The current medical challenge is to develop effective therapeutic strategies, because patients suffering from HFpEF have symptoms and quality of life comparable to those with reduced ejection fraction, but the specific medication for HFrEF is ineffective in this situation; for this, we must first understand the pathological mechanisms in detail and correlate them with the clinical presentation. Another important aspect of HFpEF is the diversity of patients that can be identified under the umbrella of this syndrome. Thus, before being able to test and develop effective therapies, we must succeed in grouping patients into several categories, called phenotypes, depending on the pathological pathways and clinical features. This narrative review critiques issues related to the definition, etiology, clinical features, and pathophysiology of HFpEF. We tried to describe in as much detail as possible the clinical and biological phenotypes recognized in the literature in order to better understand the current therapeutic approach and the reason for the limited effectiveness. We have also highlighted possible pathological pathways that can be targeted by the latest research in this field.

## 1. Introduction

The diagnosis and treatment of heart failure (HF) with preserved ejection fraction (HFpEF) represent one of the greatest challenges for physicians today. Due to its growing prevalence, it has been defined as an epidemic, and recent studies show us that under the same definition, heterogeneous clinical phenotypes with different physiopathological characteristics and mechanisms are found. Among the diseases that lead to HFpEF, we more frequently find arterial hypertension, atrial fibrillation (AF), metabolic syndrome, ischemic coronary disease, valvulopathies, cardiomyopathies as well as the cardiotoxic effects of drugs and toxins [1,2].

Although HFpEF has been seen as a mild condition in terms of organ damage, when it comes to treating these patients, there has been limited progress in developing an effective therapy. Perhaps this situation is due to the fact that HFpEF is a systemic syndrome with multi-organ involvement, which corroborates multiple cardiac and extracardiac physiopathological alterations [3,4,5].

HFpEF was defined by Dr. Luchi et al. in 1982, being the first group of researchers to describe typical heart failure symptoms in a group of patients with preserved left ventricular (LV) ejection fraction (EF) [6]. Recently, the European Society of Cardiology (ESC) united under the term HFpEF patients with preserved left ventricular EF (LVEF ≥ 50%) but with evidence of diastolic dysfunction or structural heart disease, classic signs and symptoms of heart failure and elevated plasma natriuretic peptide (NP) levels [1].

As with any definition of a syndrome, there are limitations, one of which is related to the lack of congestion in compensated HF. Another limitation is represented by the group of patients with HF symptoms who present abnormal hemodynamics exclusively during physical exercises [7]. HFpEF has been seen as a low-impact condition, yet patients have symptoms, signs, and quality of life not much different from those of patients with heart failure with reduced ejection fraction (HFrEF). However, recent data show us that this suffering is much more complicated than previously thought. Expert opinions support that HFpEF is rather a heterogeneous syndrome that includes different phenotypes with a spectrum of distinct, overlapping characteristics [8]. The etiology of HFpEF is unclear, and probably often multifactorial, but several culprits have been identified: microvascular lesions, low-grade systemic inflammation, and general oxidative stress (evolving in the context of comorbidities associated with endothelial dysfunction), all of these leading to myocardial remodeling and fibrosis [9]. These detrimental elements seem to participate fundamentally in the pathogenesis of the disease [8].

This narrative review attempts to provide a complex perspective on HFpEF. It begins with a detailed analysis of the definition of HF, continuing with the attempt to describe in detail the pathogenetic mechanisms behind the occurrence and progression of HFpEF. In the end, we tried to group the patients according to clinical and biological similarities called phenotypes and to analyze the existing therapeutic resources along with the new perspectives. The contribution of this paper consists in going through the entire challenge of HFpEF from definition to treatment, with the discussion of issues that we consider interesting due to their novelty (e.g., the use of artificial intelligence in the diagnosis of HFpEF and the approach of new therapeutical resources).

## 2. Definition of Heart Failure

The HF syndrome is characterized by a permanent change in the clinical status, which makes it very dynamic. These changes are determined by the underlying pathophysiological processes and are objectified by the change in symptoms, signs, and progression of the disease.

The problems related to HF begin with the definition of this syndrome [10,11,12]. Experts in the field are trying to standardize the existing definitions [10,13]. However, currently, there is no absolute consensus on whether the definition of HF should focus on the clinical syndrome or on hemodynamic aspects [14]. Thus, the need for a universal definition is emphasized by the increasing prevalence and the lack of unanimously accepted treatment strategies [15].

The challenge of defining HF also results from the desire to obtain a form easily used by both specialists and non-specialists (e.g., researchers, medical personnel, etc.). It would be preferable not to contain elements that can be subjective (e.g., symptoms) because they can create confusion. For example, the same maximal effort threshold may be considered normal by one person but unacceptable by another. On the other hand, paraclinical data should be obtained easily, with low inter-observer variability. And yet, the definition must contain quite specific data to be valid, a fact which for the definition used in the Framingham study is no longer valid in the current context [16].

Another problematic aspect is the differential diagnosis of the symptoms and signs of HF. HF can coexist with other diseases that can aggravate the symptoms exhibited by patients. This situation is all the more obvious in the case of patients with HFpEF [17]. Furthermore, there is the particular situation of patients who become asymptomatic under treatment. Can we consider them cured of HF, even if we know that although the symptoms can be improved, the cardiac structural abnormalities can continue silently to worsen [18]?

Referring to HFpEF, the problem of definition is even more acute. The data related to this category of patients are quite limited due to the small number of trials addressed to them. Under these conditions, the guideline recommendations include results obtained from trials that use different parameters for the included patients [16]. These differences concern LVEF whose cutoff value is over 40–45%, not 50%; in addition, there is no standardization on the cutoff values of BNP or NT-proBNP. There is a category of patients in whom LVEF improves under treatment, reaching more than 40%. These patients are categorized as patients with “improved” or “recovered” LVEF, HFpEF (borderline) or HFpEF [16]. There are no discussions related to these patients and whether they are eligible for inclusion in studies targeting patients with HFpEF. An important aspect in the clinical presentation, evolution, and treatment of patients with HFpEF is the presence of comorbidities, but precisely these patients are usually excluded from trials [16]. Another problem encountered in the interpretation of the data obtained from the studies is the lack of coherence between the inclusion criteria and the criteria that analyze the results. For instance, the levels of NPs are used as an inclusion criterion but do not represent a criterion for evaluating the success of the studied therapies [19].

### 2.1. The Role of Biomarkers in Defining Heart Failure

HF guidelines refer to B-type natriuretic peptide (BNP) and N-terminal pro-B-type natriuretic peptide (NT-proBNP). The increased level supports the diagnosis of HF, having a special value if the clinical presentation is uncertain [20]. In general, plasma BNP and NT-proBNP values are reasonably correlated. Either can be used in the diagnosis and follow-up of patients, as long as there is no confusion between their absolute values and the cutoff points [20]. It is also important to use different cutoff values depending on the type of patient (e.g., ambulatory or emergency). The ESC guideline specifies for BNP that the cutoff values are ≥35 pg/mL in outpatients, respectively ≥100 pg/mL in the case of cardiac decompensation. Similarly, for NT-proBNP, the cutoff values are ≥125 pg/mL in outpatients, respectively ≥300 pg/mL in the case of cardiac decompensation [16]. The Japanese Heart Failure Society (JHFS) specifies other cutoff values (≥100 pg/mL for BNP and ≥400 pg/mL for NT-porBNP) [11]. At the same time, the American College of Cardiology Foundation/American Heart Association (ACCF/AHA) guidelines do not specify cutoff values for these biomarkers [21,22,23].

However, NPs were not included in the definitions of HF until 2021. This may be due to certain limitations of their use. In some categories of patients, the level is modified in the absence of HF. Chronic kidney disease, aging, obesity, pulmonary embolism, atrial fibrillation, and pericardial disease are conditions that modify the levels of NPs and must be taken into account when the cutoff values are established [22]. In addition, the change in the level of NPs and NT-proBNP can be different considering the fact that BNP, but not NT-proBNP, is the substrate for neprilysin [18].

### 2.2. Universal Definition of Heart Failure

**Figure 1:**
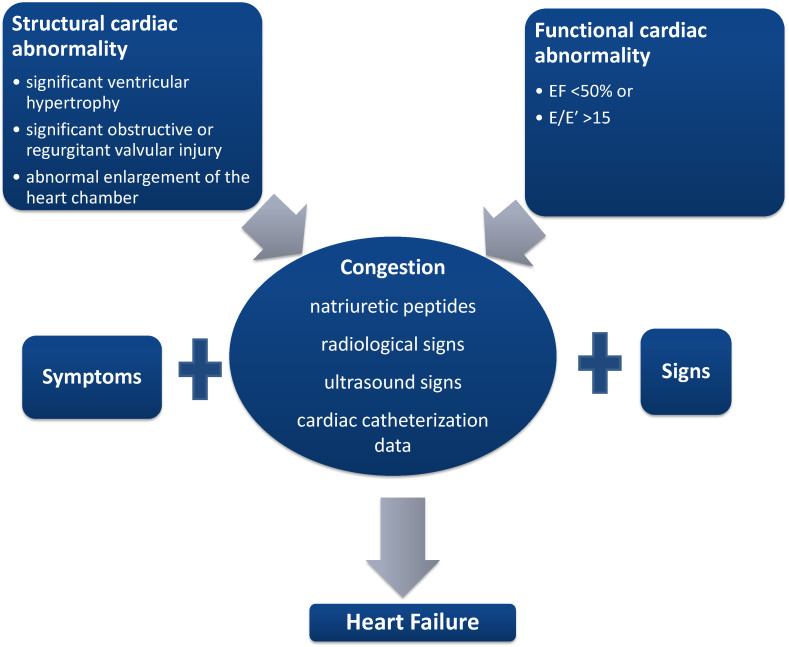
Conceptualization of the definition of HF 3. Prevalence and Demographics.

As a result of the shortcomings exposed above, responsible medical societies around the world proposed a new definition for HF, a definition that would be easy to apply in medical practice. This definition has three key points. The first key point concerns the presence of symptoms: “previous or current symptoms and/or signs caused by a cardiac structural and/or functional abnormality” [16]. This definition solves the problem of patients who become asymptomatic under treatment and directly links the clinical presentation to heart disease. The second key point concerns the detailing of objective cardiac changes evidence being mainly obtained through the use of cardiac ultrasound (LVEF < 50%, significant ventricular hypertrophy, significant obstructive or regurgitant valvular injury, abnormal enlargement of the heart chamber, E/E′ > 15) (Figure 1). And, the third key point refers to the corroboration of the first two with the presence of congestion, either by dosing NPs or by using radiological, ultrasound signs, or data obtained as a result of cardiac catheterization. An important mention refers to the fact that congestion does not need to be documented at rest; it is enough to be present during exertion [16] (Figure 1).

In the same year when the universal definition of HF was published (2021), the ESC HF guideline was updated, incorporating elements of pathophysiology, terminology, and classification. The classification of HF using LVEF largely respects the classification proposed by the ACCF/AHA in 2013, also found in the joint document issued by the Heart Failure Society of America, Heart Failure Association of the European Society of Cardiology, and JHFS under the title Universal Definition and Classification of Heart Failure. However, although the joint document refers to the stages of HF evolution, similar to the 2013 ACCF/AHA classification, which we also find in the JHFS guideline published in 2017, the ESC guideline does not mention or debate this topic. Moreover, the treatment in the ESC guide is conducted based on the classification according to LVEF, while ACCJ/AHA recommendations are addressed to the HF stages. JHFS treatment recommendations are also detailed according to LVEF [11].

## 3. Prevalence and Demographics

Referring to the general population, the prevalence of HF is estimated at 1.1–5.5% [5]. It is difficult to assess the proportion of HFpEF in the total number of HF cases due to differences in the definition, as well as in the study settings. Epidemiological studies and registries that included assessments of LVEF have estimated the proportion of HFpEF to be between 19% and 55% of all HF cases [24]. One thing is certain, we are witnessing an increase in the prevalence of HFpEF as the Framingham study shows. The authors of the study concluded that during three decades of follow-up, there was an increasing trend in the proportion of HFpEF (defined as LVEF ≥ 50%), from 41% in the period 1985–1994 to 56% in the period 2005–2014 [25].

The global prevalence of HF is estimated to be between 30 and 64 million people [5,6,7,8,24,25,26,27]. Other epidemiological analyses show that HF contributed to 1 in 8 deaths in 2017, this contribution being due to its rapid increase in incidence and prevalence [28]. In addition, this rapid growth is also constant, with a sustained rate of 1% per year, making it plausible that HFpEF will become the most common form of HF [29]. Currently, of the total number of patients suffering from HF, the proportion of those with HFpEF represents approximately one-third to one-half [8,26,28].

As expected, referring to the physiopathogenetic mechanisms, the elderly have the highest rate of HFpEF. The currently available data show that in the population over 65 years of age, patients diagnosed with HF have a normal ejection fraction in proportion to over 70% [30]; instead, if we refer to the younger group of patients (under 65 years old), data show that the proportion of those with HFpEF is only 40% [31,32]. Beyond the analysis according to age, if we refer to races and ethnicities, the statistical data show similarities between the incidence rates [33], differences being recorded according to gender, HFpEF affecting more women than men, a conclusion that suggests that gender can have a determining role in the development and progression of the disease [34]. Analyzing the risk of death in HFpEF patients, compared to the group of patients diagnosed with HFrEF, even after adjustment for age, gender, and etiology of HF, it was found that the risk is lower in the first group, thus creating the impression that HFpEF is a mild condition. However, absolute mortality is still high among patients with HFpEF, requiring intense medical efforts to reduce it [35].

It is normal to expect an increase in prevalence over time, one of the explanations being the aging of the population, another being given by the increasing prevalence of risk factors for HFpEF together with comorbidities related to HFpEF. Thus, the correct attitude is to increase awareness and improve diagnostic accuracy.

## 4. Etiology

The etiological factors involved in the development of the two pathologies (HFpEF and HFrEF) seem to be different [33]. Although the medical community is increasingly recognizing a particular pathophysiology, at present, the subtle mechanisms that produce HFpEF remain largely unelucidated.

Referring to comorbidities, in patients with HFpEF, there are frequently reported arterial hypertension, valvulopathies, and atrial fibrillation and less frequently, compared to patients with HFrEF, myocardial infarction, or left bundle-branch block (BBB) [33]. Comparing the two groups of patients, differences were also noted regarding serum potassium level and resting heart rate, which are lower in the group with preserved ejection fraction. Instead, this group has higher blood pressure values [33]. Several studies concluded that the typical patient diagnosed with HFpEF is usually an elderly woman suffering from arterial hypertension [35,36]. Anyway, in the last half-century, there has been a change in the epidemiological paradigm from smoking and uncontrolled hypertension (risk factors for ischemic heart disease—CAD and left ventricular hypertrophy—LVH) to an explosion of morbid obesity, with or without associated metabolic syndrome, diabetes mellitus, atrial fibrillation, all associated with an aging population [26]. Both age and comorbidities appear to play an essential role in the pathophysiology of HFpEF.

Comorbidities frequently associated with HFpEF are as follows: metabolic syndrome or its components (obesity, diabetes mellitus, hypertension), atrial fibrillation, diseases of the respiratory system (chronic obstructive pulmonary disease and sleep-disordered breathing), kidney disease, and anemia [37,38,39,40,41,42,43,44,45]. Glucose metabolism disorders and aldosterone excess favor both cardiac structural damage (cardiac hypertrophy and fibrosis leading to diastolic dysfunction and high ventricular filling pressures) and extracardiac comorbidities associated with HFpEF (diabetes mellitus with marked insulin resistance, severe obesity, vascular dysfunction, and pulmonary hypertension) [46]. There are recent data that show that excess accumulation of adipose tissue at the epicardial level is a risk factor for HFpEF. This conclusion is due to the association of excess epicardial fat with the presence of atrial dilatation, left ventricular hypertrophy, and diastolic dysfunction, structural characteristics considered typical for patients with HFpEF [47].

In the attempt to find the central, pivotal element of the physiopathological mechanism of HFpEF, the hypothesis of an inflammatory state was issued. This is triggered by metabolic changes induced by obesity and diabetes together with hypertension, a fact that promotes inflammation that leads to altered cardiomyocyte metabolism, coronary microvascular dysfunction, and finally subendocardial ischemia [48]. Data show that this low-grade systemic inflammation is triggered and subsequently maintained by tumor necrosis factor (TNF)-alpha together with transforming growth factor-beta 1 (TGF-beta1) [49,50]. However, there are clinical data that do not converge towards this theory. The physiopathological relationship can be bidirectional in the sense that the systemic inflammation itself can be produced by microvascular dysfunction, and it can be present before the appearance of clinical symptoms [50,51].

Although one of the blamed causes of diastolic dysfunction is myocardial fibrosis, the severity of the deterioration of diastolic function in HFpEF does not correlate with the degree of diffuse myocardial fibrosis [52]. In addition, the intrinsic phenotype of cardiomyocytes is distinct in HFrEF and HFpEF, which may explain the etiopathogenetic differences. Curl et al. show in their model of HFpEF, represented by the hypertrophic heart, that the activity of myocytes takes place under the conditions of an increased intracellular calcium (Ca^2+^) concentration, especially by increasing the current of the L-type calcium channel, a contradictory fact compared to the heart with HFrEF, where a constant reduction of the cytosolic Ca^2+^ concentration is described [53]. Research has failed to validate HFpEF-specific mechanisms; one of the few molecules associated with the onset of HFpEF, but not HFrEF, was the plasminogen activator inhibitor (PAI)-1 [54]. Among the effects attributed to PAI-1 is the impairment of metabolism that promotes the accumulation of visceral fat, senescence, and finally aging [53].

The lack of distinct mechanisms should make us understand that HFpEF is not a disease in itself, but a multifaceted clinical syndrome, characterized by heterogeneous clinical manifestations, encumbered by many comorbidities and systemic multiorgan damage.

## 5. Pathophysiology of HFpEF

### 5.1. Left Ventricular Structure and Remodeling

The initial model indicated for HFpEF in descriptive studies was that of a ventricle of normal size but with hypertrophied walls (concentric left ventricular hypertrophy) [55]. Like any model, this one was not representative of all patients with HFpEF, some of them not having cardiac structural remodeling, the left ventricular geometry being normal. However, most patients with HFpEF correspond to the previously exposed pattern, recognized by the following characteristics: hypertrophy by increasing left ventricle (LV) wall thickness and/or LV mass and end-diastolic volume within normal or near-normal limits. Hypertrophy can be concentric hypertrophy by increasing the ratio of myocardial mass to cavity volume or generalized hypertrophy by increasing relative wall thickness (RWT) [56]. There is also a group of patients who present eccentric hypertrophy, and their proportion can reach 16% [57]. Regarding the ethnic/racial variation found in patients with HFpEF at present, the studies provide little data, which does not allow us to draw conclusions.

Going further, at the microscopic level, there are differences in the structure of the cardiomyocytes of patients with HFpEF compared to patients with HFrEF, with the cardiomyocytes of patients with HFpEF being thicker and less elongated [55]. However, according to Dao-Fu Dai et al., we must take into account the fact that there is an age-dependent increase in the thickness of the left ventricular wall. This was shown by the analyses from the Framingham Heart Study and the Baltimore Longitudinal Study on Aging, studies that investigated apparently healthy adults using cardiac ultrasound. The conclusion was that there was an increased prevalence of left ventricular hypertrophy with age in both men and women, even in the absence of clinical hypertension, the most common risk factor for CVD [58]. And as aging is one of the most important contributors to HFpEF, left ventricular hypertrophy can be present but not necessarily in relation to HFpEF [58].

### 5.2. Left Ventricular Diastolic Dysfunction

The generally accepted definition of diastolic dysfunction states that the left ventricle is unable to fill to an adequate level, correlated to the body’s needs (end-diastolic volume—EDV) under conditions of low (but normal) pressure [59]. Although initially HFpEF was named as HF with diastolic dysfunction, diastolic dysfunction is not superimposed with HFpEF [59]. As Borlaug BA et al. state, diastolic dysfunction is independent of normal ejection fraction of the left ventricle. Diastolic dysfunction is the result of an abnormal distensibility of the LV that results in reduced relaxation and filling, regardless of whether or not the contractile function of the LV is normal, or whether or not these abnormalities produce symptoms [59]. It is accepted that diastolic dysfunction is part of normal human aging. The fact is reinforced by its detection in many people who do not have or will never have HFpEF. This occurs as a result of reduced filling of the LV in early diastole. This phenomenon becomes visible with increasing age in both sexes, through the decrease in ventricular elasticity due to the fibrosis of the LV walls and through the delay in active ventricular relaxation. Several mechanisms contribute to diastolic dysfunction like delayed relaxation due to reducing the efficiency of calcium capture and retention in the myocardial sarcoplasmic reticulum (SERCA2a), thus contributing to low sucking capability of the ventricle and wall rigidity. The development of diastolic dysfunction, in patients with HFpEF, does not affect the final filling volume of the LV, but this filling is difficult, as abnormally high filling pressures are required [55].

### 5.3. Ventricular Dyssynchrony

Ventricular dyssynchrony is defined as an increase in the time difference between the contractions of the two ventricles. The desynchronization of the moment of contraction of the two ventricles reduces the cardiac efficiency in performing the contraction and relaxation of the myocardium and can be correlated with the occurrence of HF [60]. It is known that cardiac dyssynchrony is, in the case of HFrEF, associated with a higher risk of adverse outcomes [61]. Although the electrical asynchrony in the case of HFpEF does not have as its main mechanism the bundle-branch block as we see in the case of HFrEF, these patients nevertheless tend to have wider QRS complexes, in these conditions, mechanical systolic and diastolic asynchrony being quite frequent [60,62]. To assess dyssynchrony, 2D speckle-tracking echocardiography (STE) is used to calculate global longitudinal strain. The advantage over the Doppler evaluation is its angle independence [63]. Patients with HFpEF have greater ventricular dyssynchrony compared to healthy people; this was expected, but dyssynchrony exists even in patients with a narrow QRS complex and LVEF ≥ 55% [62]. In HFpEF, mechanical dyssynchrony depends on QRS width (electrical dyssynchrony), ventricular hypertrophy, and diastolic but not systolic dysfunction [62].

### 5.4. Atrial Dysfunction and Atrial Fibrillation

Atrial fibrillation is a more frequent pathology in patients with HFpEF compared to those with HFrEF [64]. The left atrium (LA) functions as a buffer, absolutely necessary between the pulmonary veins and the LV. It is characterized by four functions. Two of them are passive and aim at blood storage, the reservoir function, respectively blood transfer into the LV, the conduit function. The other two are active, being represented by the accumulation of potential energy in the form of pressure, the battery function, respectively the contractile function that increases LV stroke output [65,66].

Studies have shown that once HFpEF is developed even at an early stage, patients need left atrium contraction to achieve normal LV filling compared to healthy individuals [65]. To maintain adequate LV filling, the atrial contribution, through contraction, becomes increasingly important with aging, but sustained atrial contraction also increases atrial pressure, which leads over time to atrial hypertrophy, which is a risk factor for atrial fibrillation [58]. The more important factor is the contractile function of the left atrium as patients with HFpEF, who develop atrial fibrillation, have a reduced quality of life due to reduced exercise capacity and the development or worsening of right ventricular (RV) dysfunction and increased mortality regardless of the severity of HF [67]. Melenovsky et al. found that the occurrence of atrial fibrillation in patients with HFpEF, compared to those in sinus rhythm, was associated with greater hemodynamic suffering of the right heart manifested by higher pressures in the pulmonary artery (PA), dilation and functional alteration of the right cavities [68].

### 5.5. Right Ventricle Dysfunction (RVD) and Pulmonary Vascular Disease

HfpEF is frequently associated with pulmonary hypertension (PH), right ventricular disease being part of this constellation of diseases included in the HfpEF syndrome. Up to two-thirds of patients with HfpEF present simultaneously PH [69]. Beyond the simple presence of PH, its severity is very important for a patient’s prognosis, as it is known that there is an increase in the relative risk of mortality of 28% for every 10 mmHg increase in PA pressure [70]. Reducing pulmonary arterial pressure, by using diuretics, decreases the number of hospitalizations for decompensated HFpEF [70].

RVD in most cases is caused by impaired pulmonary circulation, the most frequent cause being PH. Any increase in pulmonary arterial resistance requires an increase in myocardial contractility from the RV side, this increase being up to five times compared to the normal hemodynamic situation [71]. The problem is not that the afterload increases, but that it increases persistently, the RV not being able to cope with an increased pressure regime for long periods, given that the thickness of the RV wall is much reduced compared to that of the LV. As a result, the persistence of an increased arterial resistance will lead to RV dilation, decreased myocardial contractility (ventriculo-arterial decoupling), and decreased RV ejection fraction (RVEF). As a consequence, the RV cannot maintain an adequate cardiac output, resulting in the clinical appearance of HF.

Statistical data show that RVD can be found in up to 50% of patients with HFpEF, the development of RVD in patients with PH being a strong marker of increased morbidity and mortality, independent of the severity of HF [72].

### 5.6. Pericardial Restraint

The pericardial sac contributes to the good functioning of the heart through multiple roles, one of them being the limitation of the distension of the ventricular filling; this correlated with the venous return contributing to the increase in the intracardiac pressure [73]. Increased filling pressure in the LV is responsible for most of the symptoms in HFpEF. These elevated LV filling pressures occur in HFpEF predominantly due to myocardial relaxation abnormalities, but an important contribution also comes from pericardial constriction [74]. In an attempt to reduce intraventricular pressure, experiments were performed on animals in which it was demonstrated that pericardial resection reduces the increase in LV filling pressures in conditions of volume overload, both in normal hearts and those with diastolic dysfunction [73].

There are two phenotypes of HFpEF in which the pericardium contributes to the development and worsening of HF, the pulmonary hypertension phenotype and the obesity (cardiometabolic) phenotype [75,76]. In the case of patients with PH, it is understandable that the tension of the pericardial sac leads to an increase in RV pressure in exaggerated afterload conditions. In the obese phenotype, the combination of excess pericardial fat and increased cardiac volume leads to exaggeration of pericardial restraint [74].

### 5.7. Vascular Stiffness and Endothelial Dysfunction

**Figure 2:**
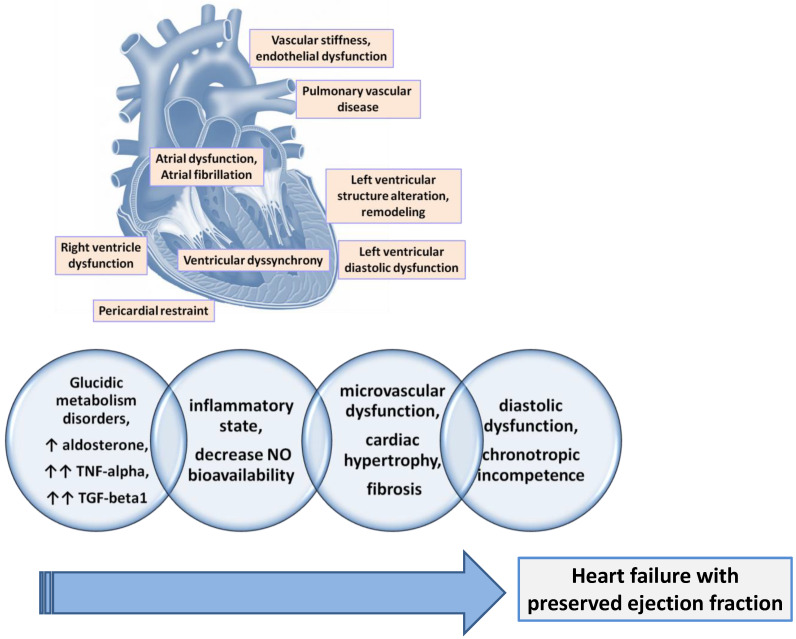
The main physiopathological mechanisms involved in the development of HFpEF.

Patients diagnosed with HFpEF frequently associate with reduced central aortic compliance and increased peripheral arterial stiffness [77]. This was expected as long as the increase in arterial stiffness is associated with diastolic dysfunction, with more arterial stiffness being responsible for the accelerated development of diastolic dysfunction [78]. Beyond the increased arterial stiffness, a sign of systemic vascular dysfunction, more than 70% of patients with HFpEF also associate with a functional coronary impairment manifested by a reduced coronary myocardial flow reserve [79]. The common element of the two vascular alterations (micro- and macro-vascular) is endothelial dysfunction. The same endothelial dysfunction was found in patients with HFpEF [80]. It is demonstrated that the presence and severity of endothelial dysfunction in patients with HFpEF contribute to a worse prognosis due to higher rates of acute cardiovascular events, a worse quality of life due to more severe symptoms, and reduced exercise capacity [77] (Figure 2).

The mechanism of endothelial dysfunction is the known one, mediated by the decrease in available nitric oxide (NO) by decreasing the activity of nitric oxide synthase 3 (NOS3) in endothelial cells or endothelial NO synthase (eNOS). But the reduction of NO bioavailability also leads to the reduction in soluble guanylate cyclase (sGC) activity in cardiomyocytes with direct negative consequences on cGMP production but also on protein kinase activity CGMP-dependent 1 (PRKG1) and, subsequently, titin phosphorylation, which leads finally to the stiffening of the cardiomyocytes. The trigger that affects NOS3 activity in patients with HFpEF is assumed to be the low-grade inflammation frequently found in their case [81] (Figure 2).

Recent research shows that cellular senescence is the basis of endothelial dysfunction. Cellular senescence is defined as the inability of the cell to divide. Beyond the natural senescence that occurs as a result of the shortening of telomeres, there is also premature senescence caused by inflammation and oxidative stress. On the other hand, senescent cells display a secretory status that in turn induces inflammation and senescence, thus creating a vicious circle [82,83]. The secretory phenotype associated with senescence consists in the secretion of growth factors (vascular endothelial growth factor), cytokines (IL-1 and IL-8), proteases (matrix metalloproteinase), and prothrombotic factors (plasminogen activator inhibitor 1) [82]. As a result, senescent cells promote tissue remodeling through cell proliferation associated with migration and tissue invasion. This tissue remodeling occurs under conditions of inflammation and oxidative stress. On the cardiovascular system, the effects consist in the development of atherosclerosis and ischemic coronary disease [84]. More recently, Andreas B. Gevaert and colleagues described the potential role of endothelial senescence in HFpEF using an accelerated senescence mouse model [85]. They found that exposure to a high-salt, high-fat diet accelerates endothelial senescence and promotes endothelial inflammation and endothelial dysfunction; this led to HFpEF as a result of the onset of diastolic dysfunction, left ventricular hypertrophy, left atrial dilatation, and interstitial fibrosis [85].

The mechanism by which endothelial dysfunction aggravates HFpEF seems to be related to the increase in central aortic pressure that prevents stroke volume from increasing, thus inducing the inability to reduce end-systolic volume in patients with HFpEF [86]. As a result of this fact (increased end-systolic volume), patients have a higher LV filling pressure at rest, and during exercise, a lower cardiac output reserve [87]. To support this theory, there are experimental data on animals that show that LV diastolic dysfunction (assessed by echocardiographic parameters) is limited if the nitroxyl donor, 1-nitrosocyclohexyl acetate (1-NCA), is administered chronically [88]. The supplementation of nitric oxide also led to a reduced level of the pro-fibrotic signal (Connective Tissue Growth Factor—CTGF) and to a reduced size of cardiomyocytes, elements that create the premise of reducing the stiffness of the LV wall [88]. Attempts to translate the data obtained on the animal model into clinical studies aimed at improving NO bioavailability and mitigating endothelial dysfunction have failed to demonstrate significant efficacy in patients with HFpEF. However, endothelial dysfunction and HFpEF share a series of elements (increased oxidative stress, inflammation, fibrosis) that cannot be ignored [9,89]. It remains to be seen how these experimental data will be exploited and transformed into therapeutic resources in clinical trials.

### 5.8. Chronotropic Reserve

Chronotropic incompetence (inability to increase heart rate) is a common functional alteration in patients with HFpEF, its prevalence varying between 57% and 77% depending on the study [86]. The cardiac output is directly influenced by the heart rate, it being calculated as the beat volume multiplied by the heart rate. One of the causes of exercise capacity limitations in patients with HFpEF is the inability to increase cardiac output appropriately due to the inability to increase heart rate during exercise. This fact is added to the impairment of the systolic volume reserve present in patients with HFpEF [87]. There are insufficient data to explain the mechanisms leading to the impairment of the chronotropic response in patients with HFpEF, but the assessment of chronotropic incompetence (CI) is important because it is a prognostic marker for increased risk of adverse clinical events [90]. Moreover, some studies showed a direct link between CI and reduction of peak oxygen consumption (VO_2_) in patients with HFpEF, with low VO_2_ being also associated with a worse prognosis in patients with HF [91]. One of the causes of CI appears to be impaired cardiac β-receptor sensitivity [92].

### 5.9. Cardiac Aging

Data from the literature show that one of the culprits responsible for the HFpEF epidemic is the aging of the general population, a process strongly associated with the development of diastolic dysfunction. Aging cannot be avoided, it is a normal process, but it involves cardiac and vascular alterations that are found often prematurely in patients with HFpEF. These alterations have already been mentioned in this paper and are represented by diastolic dysfunction, chronotropic incompetence, loss of systolic reserve, endothelial dysfunction, and vascular stiffening [55]. They hide functional changes that occur at the cardiac and vascular levels as a result of aging. The functional changes aim at calcium metabolism with its deficient use in the myocytes, the decrease in β-adrenergic reserve, and the endothelial dysfunction related to the decrease in NO bioavailability. Physical deconditioning is added to these as a possible explanation or as a possible effect of them. The natural aging process of the heart can be accelerated by the presence of HFpEF [55], this unwanted contribution is greater in the case of women and overweight people [47].

The use of artificial intelligence (AI) in the analysis of ECG recordings made it possible to estimate the real age of the heart; this is greater than the chronological age (CA) in the case of premature cardiac senescence. This was possible using algorithms that measure PR interval, QRS duration, QT interval, and QTc interval [93]. Using this type of AI-supported algorithm, Frederik H. Verbrugge and colleagues demonstrated that premature cardiac senescence was greater in the obese [94]. This has been associated with obese patients with more frequent structural remodeling, more pronounced diastolic dysfunction, and a higher risk of atrial fibrillation [94]. Chenyu Li et al. linked obesity to inflammation as a possible mechanism for inducing cardiac senescence and HFpEF development [95]. They demonstrated the direct pro-inflammatory effects of nutrient overload. Nutrient overload can directly induce inflammation in cardiomyocytes, macrophages, and endothelial cells. Induction of inflammation seems to be based on “metabolic reprogramming”. Normally, cardiac macrophages help maintain homeostasis by removing senescent dead cells and defending against infections without inducing an immune response [96]. The metabolic reprogramming as a result of the surplus of nutrients causes macrophages to become promoters of inflammation with detrimental effects on the myocardium [95]. Metabolic reprogramming also takes place at the level of endothelial cells, this time as a result of inflammation; this reprogramming leads to the exacerbation of inflammation and the initiation of a vicious circle that leads to endothelial senescence and, in the end, endothelial dysfunction [97].

### 5.10. Hypervolemia

In HFpEF, there is no unanimous belief related to the presence of volume overload. Clinically, this overload is highlighted by the presence of edema, ascites, pleural collections, etc., signs absent most of the time in the case of HFpEF. Biologically, congestion is highlighted by measuring NPs. Even if these are increased, the magnitude of the increase is not great. In addition, their increase reflects ventricular parietal stress, not necessarily volume overload [98].

However, there is the general idea that, even in HFpEF, decompensation occurs due to congestion and volume overload, the kidneys being most likely responsible for this, patients with HFpEF presenting with a status of chronic cardio–renal suffering that involves the alteration of sodium and water homeostasis [98,99]. However, the way volume loading is presented differs between patients with HFrEF and those with HFpEF. If in those with HFrEF cardiac decompensation means more fluid in the vessels, in those with HFpEF it means more fluid in the interstitium without a significant increase in intravascular volume [100]. This fact can also explain the reduced effect of diuretics in patients with HFpEF compared to the significant effect of sodium–glucose co-transporter 2 inhibitors (iSGLT2) in improving dyspnea and reducing cardiac decompensation [100].

Judicious use of the terms “congestion” and “volume overload” is mandatory in order to use the right therapy, as congestion can occur even in the absence of volume overload [101].

## 6. Clinical Manifestations

There are no specific symptoms or signs of HFpEF. They are the same as we find in HF patients in general. In terms of frequency, dyspnea is by far the most frequent symptom reported by patients, limiting the ability to exercise. Dyspnea can manifest itself with different degrees of severity and, just like in HFrEF, it can manifest itself during exertion, at rest, or in the form of paroxysmal nocturnal dyspnea. Other symptoms shown are low exercise tolerance, excessive fatigue, chest pain, or just a feeling of pressure or chest discomfort [8]. Signs of RVD (ankle edema and jugular venous distension), along with liver pains, are often absent from the clinical manifestations of patients with HFpEF. Clinical manifestations like fatigue in the early stages of HF syndrome or loss of appetite and cachexia in late-stage HF can occur [26].

## 7. Diagnosis

**Figure 3:**
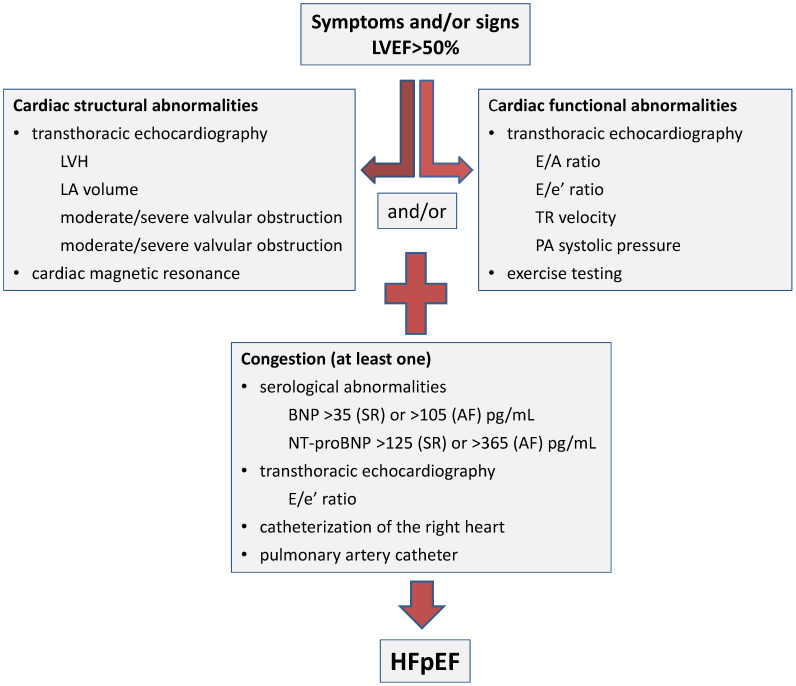
Diagnostic algorithm diagram of HFpEF.

There are no differences in the clinical manifestations depending on the HF phenotype, the manifestations in HFpEF being similar to those in HFrEF. But in patients with HFpEF, the diagnosis is much more difficult because the ejection fraction is normal, making the differential diagnosis of patients with non-cardiac dyspnea difficult. Structural (concentric left ventricular hypertrophy, left atrial remodeling) and functional (diastolic dysfunction, impairment of cardiac reserves) cardiac abnormalities are frequently present in patients with HFpEF [102] (Figure 3). Along with these, a series of systemic changes are frequently present, with variable intensity in patients with HFpEF: systemic and/or pulmonary vascular dysfunction, damage to skeletal muscles, and changes in body composition [102,103].

The definition of HFpEF according to the guidelines of the European Society of Cardiology (ESC) involves the presence of signs and/or symptoms of heart failure, in conditions of preservation of the left ventricular ejection fraction (LVEF ≥ 50%), but with evidence of either structural heart disease or diastolic dysfunction, along with increased levels of NPs [1] (Figure 3). Although HFpEF has historically been known as heart failure due to diastolic dysfunction, this pathology is absent in some cases. The evidence of diastolic dysfunction is not necessary for diagnosis as long as there are signs of cardiac structural alteration and congestion [104]. Considering the complexity and heterogeneity of HFpEF, the clinical approach is comprehensive. Several sets of clinical (patient history and physical examination), biochemical (serum level of NPs), imaging, and hemodynamic evidence are compiled for the positive diagnosis (Figure 3).

According to the guidelines of the European Society of Cardiology (ESC), the diagnosis of HFpEF is excluded in the presence of a level considered normal for BNP (BNP < 100 pg/mL). However, this approach is not valid for all patients with HFpEF. The level of BNP is a marker of LV parietal stress, which can sometimes be absent even in conditions of increased filling pressures (characteristic of HFpEF), because the hemodynamic changes, which lead to the appearance of LV wall stress, are of a smaller magnitude in HFpEF compared with HFrEF [105,106].

### 7.1. Clinical History and Physical Examination

The first steps in evaluating a patient with suspected HFpEF involve gathering data related to the clinical history and performing a physical examination. Physicians should look for very specific symptoms such as orthopnea and paroxysmal nocturnal dyspnea to support the diagnosis. Nevertheless, in practice, symptoms such as fatigue and/or exertional dyspnea are much more common; but less specific for HF. Chest pain, often reported as chest discomfort, has many causes that are independent or combined: large epicardial coronary artery disease, microvascular dysfunction, and mismatch of oxygen supply according to myocardial demand during physical activity [107,108]. Peripheral edema is a sign that suggests either the presence of isolated right heart failure or HFpEF in an advanced stage with global manifestations of HF. Physical examination can provide clues for HFpEF diagnosis. Within patients with HFpEF, 60–75% of them have a BMI over 30 kg/m^2^ [109]. Physical evaluation can detect signs of congestion such as jugular distention, peripheral edema, crackles, and also signs of left ventricular failure like gallop sounds. Complete irregular heart rhythm suggests AF [110].

### 7.2. Transthoracic Echocardiography

The main parameter that allows classifying patients into one of the forms of HF, according to the ESC guidelines, is LVEF. Although it is not a perfect parameter, mainly due to interobserver variability, the complete echocardiographic evaluation including LVEF remains the non-invasive method of diagnosis of HFpEF most used in medical practice. Diastolic dysfunction can be assessed by several parameters that are used to indirectly determine the pressures at which LV filling is achieved in the case of suspected HFpEF. For this purpose, different parameters are used, mainly evaluating the performance of the LV but also those of the RV. The following parameters are more frequently used: peak acceleration rate of mitral E velocity, ventricular filling velocity ratio (E/A), ratio between rapid ventricular filling velocity and ventricular myocardial relaxation velocity (E/e’), isovolumic relaxation time (IVRT), peak tricuspid regurgitation (TR) velocity, RV fractional area change [111]. Echocardiography can be used to determine left atrium remodeling and contractility dysfunction [55]. The assessment of LA function is more difficult to achieve, the measurement of LA strain being a method that can be useful in practice for the assessment of patients with HFpEF [112,113]. The guideline recommendation is to use LA volume in the assessment of diastolic dysfunction, the limit value being 34 mL/m^2^ [111].

#### 7.2.1. Speckle-Tracking Echocardiography (STE)

Speckle-tracking echocardiography (STE) is a method that has become accessible recently. It is more laborious, but changes in STE parameters are more subtle and are associated with cellular deficiencies (health of tubular T elements, deficient intracellular calcium homeostasis, deficiencies in excitation–contraction coupling) present at the onset of myocardial fibrosis [114,115]. With all these limitations, echocardiographic evaluation of the patient with HFpEF is crucial.

#### 7.2.2. Automated Echocardiographic Detection of HFpEF

With all these limitations, the echocardiographic evaluation of the patient with HFpEF is crucial but time-consuming and sometimes with incomplete results that require additional, expensive, and invasive investigations. Recent advances in the use of artificial intelligence in the field of video data interpretation offer great promise that computational methods can better interpret ultrasound images. Ashley P. Akerman and colleagues developed an AI model for the diagnosis of HFpEF. This model based on the interpretation of an ultrasound recording demonstrated better discrimination than clinical scores of patients with or without HFpEF. In addition, it identified patients with a higher risk of mortality [116]. Beyond obtaining an accurate and rapid diagnosis, the use of unsupervised machine learning helps to identify physiologically and prognostically distinct subgroups using the assessment of diastolic function by Doppler parameters [117].

### 7.3. Cardiac Magnetic Resonance

For the structural but also functional evaluation of the heart, in many respects, cardiac magnetic resonance (CMR) is the gold standard. It allows accurate measurement of volumes, mass, and ejection fraction for both ventricles. Using techniques of late gadolinium enhancement (LGE) together with T1 mapping, CMR allows the identification and characterization of myocardial fibrosis, a fact that recommends it as the method to be used in identifying the etiology of HF [118]. By characterizing the myocardial tissue, CMR brings important data that can support the etiology of HFpEF in the case of myocarditis, sarcoidosis, amyloidosis, and Fabry disease [119]. In addition to LVEF, using fast long-axis strain for assessing left ventricular longitudinal function showed effectiveness in all HF phenotypes [120]. Magnetic resonance elastography (MRE) uses mechanical shear waves to quantitatively assess the stiffness of tissue and can be used as a marker for patients with HFpEF [66]. The use of CMR also brings benefits in the accurate measurement of LA size and volume.

### 7.4. Exercise Testing

Exercise testing is an investigation reserved for patients who develop abnormalities of the ventricular filling pressure only during exercise, while at rest they are normal, the validation of these abnormalities being necessary for the diagnosis of HFpEF. The use of exercise testing also involves catheterization of the right heart to detect exercise-induced hemodynamic changes [121]. Invasive exercise tests are the only ones capable of directly evaluating the hemodynamic parameters that define HFpEF, increased pulmonary capillary pressure, and cardiac output reserve. Therefore, they represent the gold standard test for the diagnosis of HFpEF [105], but invasive exercise testing needs operator expertise and is expensive. Instead, due to its accessibility, exercise echocardiography represents an alternative to invasive stress testing [122]. The use of exercise echocardiography as an alternative method is still under debate, with studies showing contradictory results [123,124,125].

**Figure 4:**
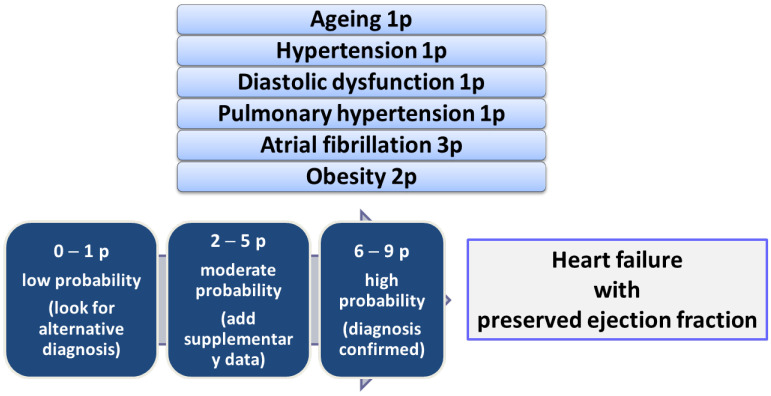
H2FPEF score used for risk assessment of HFpEF—the parameters used, the reference intervals, and the significance of each of them.

We can identify patients with suspected HFpEF using the H2FPEF score (Figure 4). This score is giving 2 points for BMI > 30 kg/m^2^, 1 point for two or more antihypertensive drugs, 3 points for paroxysmal or persistent atrial fibrillation, and 1 point for each of the following: echographic pulmonary artery systolic pressure > 35 mmHg, age over 60 years and E/e’ ratio > 9. The maximum score is 9. There are three intervals of probability of the presence of HFpEF. The first includes a score of 0 or 1, which indicates a low probability (25%), the existence of alternative causes for the symptomatology, allowing the diagnosis of HFpEF to be excluded. The second score range, between 2 and 5, indicates a moderate probability (40–80%), additional data being required for a positive diagnosis. The third score interval (>6 points) confirms the diagnosis of HFpEF, the probability being over 90% [126] (Figure 4).

## 8. HFpEF Phenotypes

**Figure 5:**
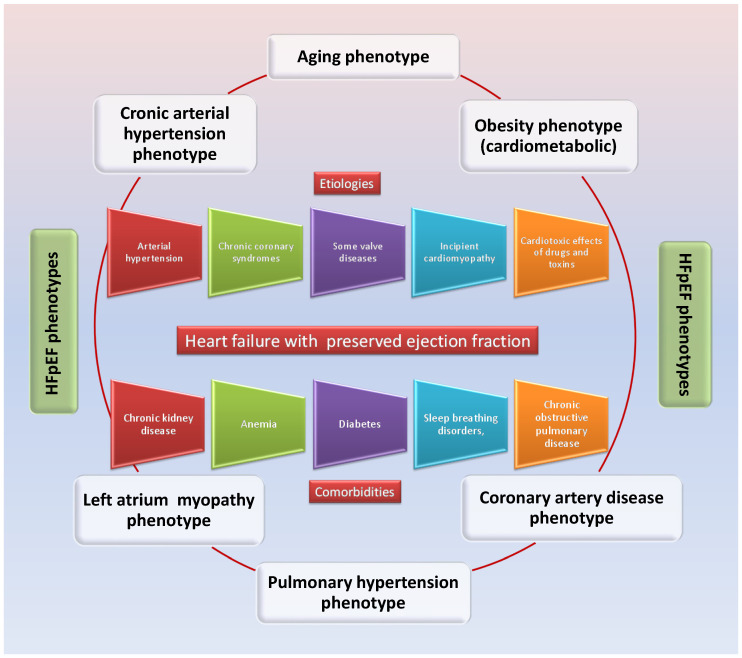
HFpEF phenotypes. Etiology and comorbidities responsible for the development of HFpEF phenotypes.

The definition of HFpEF phenotypes is not homogeneous among researchers, in part due to the different understanding of the molecular mechanisms responsible for the multiple facets of the disease. This creates difficulties in integrating the results obtained in various studies, a fact that represents a major obstacle in obtaining effective therapies. There are six major phenotypes of HFpEF described, characterized by distinct clinical features [127,128] (Figure 5):

- The phenotype characterized by the aging processes (aging phenotype);
- The phenotype characterized by the excess presence of adipose tissue (obesity or cardiometabolic phenotype);
- The phenotype associated with arterial hypertension;
- The phenotype associated with pulmonary arterial hypertension;
- The phenotype associated with ischemic coronary disease (the phenotype of coronary artery disease);
- The phenotype associated with left atrial myopathy.

### 8.1. Aging Phenotype

The direct relationship between age and HF is a certainty if we look at the epidemiological data, which show that advanced age is one of the most important contributors to the increase in the prevalence of HFpEF, both directly and through the number of comorbidities associated with advanced age and the frailty of the elderly patient [129,130]. There is certainly a direct proportional relationship between the prevalence of HFpEF and the age of the patients when we talk about the group of elderly and very elderly patients (≥80 years) [131]. And this can be explained by the systemic changes induced by aging, along with the cardiac structural changes that produce functional alterations and, finally, HFpEF [132]. There are data showing an age-dependent decline in key growth signaling pathways, including insulin-like growth factor-1 (IGF-1) [133]. This decline was associated with a higher risk of developing HF in the elderly population without heart disease [134]. An explanation of this association, age–HF, can be given by the fact that cardiomyocyte activity deteriorates rapidly in the presence of mitochondrial alterations, these alterations occurring under conditions of increased reactive oxygen species (ROS) production and inadequate detoxification, which leads to the onset of cardiomyopathy in elderly patients [135]. Another mechanism is related to the structure of the extracellular matrix (ECM). It is composed of complex proteins, synthesized by cardiac fibroblasts, located around cardiomyocytes with the role of providing structural and biological support. These proteins include collagen, elastin, fibronectin, and laminin [136]. The alteration (qualitative or quantitative) of the ECM structure leads to an increase in the stiffness of the cardiac wall and to the development of diastolic dysfunction [137]. Simultaneously with the alteration of the ECM structure, the impairment of the active relaxation of cardiomyocytes also contributes to the development of diastolic dysfunction. This happens due to defective Ca^2+^ cycling and reduced Ca^2+^ sensitivity of myofilament proteins that may lead to dysregulation of cardiomyocyte relaxation [138,139].

Research on microRNAs (miRNAs) shows that they are involved in senescence and cardiovascular disease, due to an important regulatory role [140,141]. Added to this is the impairment of the regenerative capacity of cardiac stem cells, both due to the senescence of stem cells and the unfavorable conditions of development that may appear in their microenvironment, due to advanced age [142]. Aging changes include changes in the structure and shape of the myocardium (brown atrophy of the myocardium, focal amyloid deposits, sigmoid-shaped ventricular septum) as well as the surrounding structures (increase in subepicardial fat) [143]. Muscle wasting is a strong predictor of frailty and reduced survival in patients with HF [144].

Age-related changes include a proinflammatory state (highlighted by important levels of TNF-alpha, monocyte chemoattractant protein, and ROS) and neurohormonal dysregulation (increase of angiotensin II and endothelin levels). Experimental studies show that exposure to angiotensin II (Ang II) affects both cardiomyocytes (inducing hypertrophy) and the extracellular matrix (increasing the degree of fibrosis), ultimately leading to impairment of cardiomyocyte relaxation [145]. Age also affects the vascular system, at the arterial level producing an increase in stiffness, resulting in a widening of pulse pressure and systolic arterial hypertension [146,147]. The mechanisms that increase peripheral arterial resistance are as follows: inflammation (via cytokines and oxidative stress) that mediates the breakdown of collagen; neuroendocrine activation (via Ang II and aldosterone) with consecutive fibroblastic activation; alterations in glucose metabolism (hyperglycemia and hyperinsulinemia or insulin resistance development) leading to the production of advanced glycation end products [148,149]. Arterial stiffening, by increasing pulse wave velocity (PWV), leads to an increase in central arterial pressure and, consequently, to an increase in ventricular afterload. This afterload mismatch will lead to decreased cardiac output reserve and ventriculovascular uncoupling [150].

In these circumstances, it is not surprising that studies have shown that aging is one of the strongest risk predictors of HFpEF [151].

### 8.2. Obesity Phenotype (Cardiometabolic)

Along with advanced age, increased body mass index represents another important risk factor, recognized to be involved in the new onset of HFpEF [9,34]. In the US, more than half of patients with HF have HFpEF with signs of obesity and heterogeneous metabolic syndrome (insulin resistance, diabetes mellitus, hypertension, and hyperlipidemia) [152,153]. One of the mechanisms by which obesity leads to HF is the increase in arterial stiffness, central obesity along with age and arterial hypertension being major determining factors of arterial stiffness [154]. Another element responsible for the presence of HFpEF in obese people is obstructive sleep apnea, a pathology with a high prevalence among them. There are multiple mechanisms implicated in the pathogenesis of HFpEF, triggered by obstructive sleep apnea through hypoxia. Hypoxia induces excessive sympathetic activation responsible for increasing systemic blood pressure (increasing LV afterload) and triggering atrial or ventricular arrhythmias. In addition, it inflicts hypoxic pulmonary vasoconstriction that increases RV afterload and reduces LV preload as well as the increase in oxidative stress that maintains inflammation [155,156]. Obese patients with HFpEF exhibit particular pathophysiological features, including increased epicardial fat mass, greater hypervolemia, greater biventricular hypertrophy, and abnormal RV–PA coupling [107]. There is solid evidence that excess epicardial adipose tissue represents an independent risk factor for HFpEF, it being actively involved in the pathophysiology and progression of HFpEF [157].

### 8.3. Hypertension Phenotype

As already mentioned in this paper, we cannot talk about HFpEF without involving hypertension, it being recognized by all research as a major risk factor [28]. This statement is the result of prevalence data, among the population diagnosed with HFpEF, with the prevalence reaching up to 90% in certain studies [158].

The mechanisms are complex and complicated and aim to change the structure of the ventricular wall as well as the structure of the arterial wall at the macro- and micro-vascular levels. The ventricular wall is affected by hypertrophy and fibrosis as well as a result of adaptation to an increased afterload, which leads to diastolic dysfunction. Alteration of the arterial wall induces stiffening with direct effects on the progression of HFpEF [28,158].

### 8.4. Pulmonary Hypertension Phenotype

Pulmonary venous hypertension resulting from a long-term increase in left atrial pressure is the most common etiology of pulmonary arterial hypertension (PH) [159]. There are even data that the prevalence of PH is even slightly higher in patients with HFpEF than in those with HfrEF [160]. Beyond PH secondary to left heart disease, there is also an arterial component, which must be suspected in patients with excessively high PH values, disproportionate to the degree of severity of left heart damage, in which there is a combination of pre- and post-capillary mechanisms. This arterial component depends on genetic factors that influence arterial remodeling through the hypertrophy of vascular smooth muscle cells and the deposition of extracellular matrix, but also vascular reactivity by altering the balance between endothelin and nitric oxide [159]. There are certain characteristics of patients that associate HfpEF with RVD, as shown by Melenovsky et al. that patients with HFpEF and RVD are more frequently men, have greater congestion, a fact highlighted by higher levels of natriuretic peptides, more frequently have atrial fibrillation and ischemic coronary disease, all of which, in the end, place them in a worse functional class [68].

### 8.5. Coronary Artery Disease (CAD) Phenotype

Compared to arterial hypertension, the prevalence of CAD is lower but still at alarming levels, with the HFpEF registries recording prevalence rates of up to 50% among these patients. There are inter-ethnic differences in terms of prevalence, this being higher among Caucasians [161]. The presence of CAD in patients with HFpEF means that they have a greater risk of deterioration of LV function related to ischemic mechanisms, which implies a poorer prognosis compared to patients with HFpEF without CAD [162]. Beyond the possibility of epicardial coronary damage, patients with HFpEF also exhibit functional microvascular dysfunction, with the same risk of aggravation of the contractile function of the LV [162].

### 8.6. Left Atrium (LA) Myopathy Phenotype

Starting from the important role that the left atrium has in the proper functioning of the LV, a new phenotype of HFpEF was defined by Patel et al. related to LA suffering. Thus, several conditions have been defined that vary from preserved atrial function to LA myopathy [163]. The presence of LA myopathy, independent of AF, correlates with reduced ventricular performance (lower stroke volume reserve) and alterations in the arterial systems (a higher pulmonary vascular resistance with higher systolic pressure in the pulmonary artery and a flow reserve lower coronary) [69]. Atrial fibrillation (not necessarily an expression of LA myopathy) and HFpEF share common pathophysiological features (including a relative deficiency of nitric oxide) and common symptoms [164]. Manifestation of LA myopathy is not necessarily related to atrial fibrillation, this being highlighted, in this phenotype, by the fact that the restoration of sinus rhythm may not completely alleviate the symptoms. Therefore, among patients with AF and exaggerated dyspnea, we must look for the possibility of the existence of HFpEF, the LA myopathy phenotype, which can be present with a probability of 50% [165], a fact also evoked by the inclusion of AF in the H2FpEF prediction score (Figure 4). There are other classifications depending on the hemodynamic status, the type of myocardial injury reflecting the heterogeneity of patients with HFpEF, and the difficulty in classifying them under the same “umbrella”.

It is reasonable to believe that different HFpEF phenogroups may have different geographic distributions, making it difficult to standardize statistical data. In Asia, patients with a mild hypertensive profile are more common than in the Western countries (hypertrophic, rigid hearts) [166,167].

## 9. HFpEF Biological Phenotypes

In an attempt to achieve a better classification of patients with HFpEF, a classification based on biological markers was attempted, the advantage being a better overlap in the pathogenetic mechanisms of HFpEF. For now, there are four identified biological phenogroups [166,168].

### 9.1. Natriuretic Peptide Deficiency Syndrome

This phenotype is found in young patients, with a relatively normal natriuretic peptide value and showing moderate diastolic dysfunction. Lower natriuretic peptides are also observed in obese HFpEF patients due to enhanced pericardial restraint (similar to constrictive pericarditis) [107].

### 9.2. Excessive Activation of Plasminogen Activator Inhibitor (PAI)-1

The available data show that this protein, in increased quantity, has significant effects on the occurrence and progression of diseases of the cardiovascular system due to the promotion of metabolic syndrome, atherosclerosis, and cardiac fibrosis. PAI-1 production is stimulated by angiotensin II in adipocytes. This phenotype of HFpEF is characterized by inflammation and accelerated aging, in patients with multiple metabolic comorbidities [54].

### 9.3. Extreme Cardiometabolic Syndrome

This phenotype is characterized by the presence of obesity, along with its complications, diabetes mellitus, and obstructive sleep apnea [169].

### 9.4. Right Ventricle–Cardiac–Abdominal–Renal Syndrome

It is characteristic of elderly patients who associate chronic kidney disease with cardiopulmonary pathology. This phenotype is characterized by the association between increased intra-abdominal venous pressure, renin—angiotensin—aldosterone system (RAAS) activation and oxidative damage, and PH and RV dysfunction [170].

The prognosis differs between these groups, the worst being the last two described.

## 10. Phenotypes of HFpEF Identified Using Machine Learning Techniques

Considering the enormous amount of data that needs to be processed, several researchers have used machine learning techniques to identify clinical or biological phenotypes, with the ultimate goal being to obtain a personalized treatment [171,172,173]. Using machine learning techniques, subgroups of patients were obtained that combined elements of clinical phenotypes with elements of biological phenotypes [173]. Thus, Rebecca J. Woolley and colleagues identified subgroups with different pathological pathways and clinical outcomes [173].

One group characterized by the prevalence of diabetes and kidney disease had the highest plasma concentrations of creatinine, glucose, gamma-glutamyl transferase (GGT), and growth differentiation factor-15 (GDF-15). The levels of GGT and GDF-15 increase in conditions of inflammation, oxidative stress, ischemia, and mechanical injury [174,175] so this group has been associated with the activation of inflammatory pathways. Another group was characterized by the highest prevalence of ischemic etiology, and chronic lung disease presented the most symptoms, as well as the highest levels of NT-proBNP and troponins and the activation of phosphoinositide 3-kinases (PI3K). PI3K is associated with pathways involved in protein synthesis, regulation of cell proliferation, and cell survival [176]. And with all these differences between groups, the size and function of the left ventricle and the dimensions of the left atrium were similar between the groups [173]. So even if from a hemodynamic point of view the treatment can be similar, it can be personalized depending on the pathogenetic pathways involved.

## 11. Differential Diagnosis

The main differential diagnosis concerns dyspnea, the symptom that brings the patient to the physician most often, with severe dyspnea being a frequent cause of hospitalization. But dyspnea is a less specific symptom for HF; there are various extracardiac pathologies to consider [68], or situations such as physical deconditioning and obesity that induce this symptom [127]. Another problem is the assessment of dyspnea in patients with respiratory diseases, in whom there is an overlap between chronic obstructive pulmonary disease (COPD), HFpEF, and pulmonary hypertension [177]. Along with chronic lung disease, there are other conditions that produce characteristic symptoms of HFpEF; we mention here anemia, chronic kidney disease (CKD), or diseases that produce volume overload such as nephrotic syndrome, hypothyroidism, and arteriovenous fistula [178]. The signs presented by HFpEF patients are usually represented by pulmonary or peripheral congestion. In many cases, the symptoms and signs of HFpEF and HFrEF are indistinguishable.

## 12. Comorbidities

The European Society of Cardiology has developed a series of guidelines for the diagnosis and treatment of acute and chronic HF, emphasizing the importance of evaluating cardiovascular risk factors and comorbidities in HFpEF. Its recommendation is mandatory for the screening and treatment of cardiac or noncardiac causes of HFpEF, along with concomitantly diagnosed comorbidities [179]. The treatment of comorbidities is important, due to the systemic endothelial inflammation promoted by some of them, inflammation that produces functional and then structural remodeling of the cardiovascular system [180]. The most representative comorbidities (which can potentiate each other in aggravating HFpEF) are the metabolic syndrome or its components (obesity, type II diabetes) [153], respiratory system disorders (sleep breathing disorders, chronic obstructive pulmonary disease), anemia of various etiologies, and chronic kidney disease (CKD) [39,40,41,42,43,44,45]. In patients with HF, the most common non-cardiac comorbidities are CKD, anemia, diabetes mellitus, and obesity. Patients with HFpEF have a higher prevalence of comorbidities compared to those with HFrEF [1]. The most prevalent comorbidity found by Streng et al. was CKD (50%), with diabetes mellitus being present in only 45% of patients with HFpEF [81].

The central, pivotal element is the excess of visceral fat, the fat being recognized to be an active tissue, capable of producing large amounts of pro-inflammatory cytokines [181]. These, together with the increased resistance to the action of insulin, manifested by this tissue, which leads to hyperinsulinemia and hyperglycemia, produce mitochondrial dysfunction, microvascular damage, and autonomic neuropathy that cause hypertrophy, fibrosis, cardiac and vascular stiffness, and finally HF [9,182]. The development of the concept of diabetic cardiomyopathy, as a suffering of the myocardium beyond the ischemia associated with diabetes mellitus, made HFpEF to be considered an expression of this disease [183]. In the case of diabetic cardiomyopathy, there are changes in the myocardial tissue that induce a passive stiffening not observed in non-diabetic HFpEF tissue [184,185]. In diabetic patients, there was a higher prevalence of both myocardial hypertrophy and fibrosis; as in the case of patients with HFrEF, patients who associate with HFpEF and diabetes mellitus having a worse prognosis [184,185].

Iron deficiency proved to be of great prognostic importance in patients with HF. Whether it presents with anemia or not, its presence means reduced exercise tolerance and a worse prognosis [186]. The same is true for COPD, a pathology frequently associated with HFpEF, in addition, anemia in the patient with HFpEF means a higher risk of sudden cardiac death or malignancy [187].

## 13. Prognosis

Chronic HF is a syndrome characterized by a considerable loss of quality of life and a lower life expectancy, together with a high socioeconomic burden [26]. Although initially HFpEF was regarded as an incipient form of HFrEF, systematic longitudinal studies did not show the existence of an evolutionary link between the two entities, the transition from HFpEF to HFrEF being rare [188]. Considered to be a milder form of HF, with a better prognosis than HFrEF, as shown by some research studies [36,189], there are some concerns regarding the real prognosis of these patients, with some studies showing similar outcomes, prognosis, and survival between the two groups [190,191]. Somaratne et al. state a 50% better survival rate in the case of patients with HFpEF compared to those with HFrEF [188]. However, their evolution is marked by substantial morbidity and mortality, with hospitalizations for cardiac decompensation (35% at two years) and still high mortality (14% at two years) [29]. On the other hand, other studies, which evaluated patients with cardiac decompensation that required hospitalization, reported a 1-year mortality of approximately 20–30% [192]. Another large study reports a 5-year survival among patients with HFpEF, hospitalized for HF decompensation, between 35 and 40% [28]. The research concludes that patients with HFpEF have a poor quality of life comparable, for a better understanding, to patients with end-stage renal disease, requiring frequent medical interventions and hospitalizations [193]. And in perspective, if in the case of patients with HFrEF a significant improvement in survival was found in the last decade, the evolution of patients with HFpEF did not undergo significant improvements, despite the appropriate use of available therapies. The highest risk of hospitalization for cardiac decompensation or death is found among congestive patients, with excessive water and sodium retention and high plasma levels of BNP, this risk being clearly diminished in patients with HFpEF who develop an abnormal hemodynamic response only during exercise [114]. The lack of studies leading to evidence-based therapeutic strategies has been blamed for these high mortality and morbidity rates in HFpEF.

However, several prognostic factors such as B-type natriuretic peptide (BNP), tPA/PAI-1 complex, diabetes mellitus, cystatin C, and growth factor 15 (GDF-15) indicate an increased risk of unfavorable evolution in patients with HFpEF [194,195,196,197,198,199,200,201,202,203,204,205].

Comparing the two types of HF (HFpEF and HFrEF), there are notable differences in plasma concentrations of both BNP and NT-proBNP, which is understandable considering that natriuretic peptides reflect increased tensional stress in the LV wall rather than pressures of LV filling, with this tensional stress being lower in HFpEF compared to HFrEF. However, regardless of the form of HF we are referring to, measuring the level of natriuretic peptides is important, as they represent an important prognostic factor [195,196].

Other important structural and functional prognostic factors are decreased LV compliance and RV remodeling [206]. Comorbidities that worsen the prognosis are ischemic heart disease, diabetes mellitus, and chronic renal failure [207]. The presence of diabetes worsens the prognosis of patients with HFpEF by increasing the risk of sudden cardiac death, especially in those with insulin requirements [208,209], but the risk of non-cardiac death is also increased, even higher than in patients with HFrEF [210]. Analyzing data from the I-PRESERVE study, Zile et al. found that patients with HFpEF have an increased risk of death from non-cardiac causes (28–30%), sudden cardiac death (26–28%), progressive worsening of HF (14–28%), stroke (7–9%), and myocardial infarction (3–5%) [210].

## 14. Treatment

The prognosis of patients with HFpEF is generally affected by the fact that they do not respond to therapies known to be effective in patients with HFrEF [26].

### 14.1. Pharmacological Therapy

Studies conducted to date have not provided strong evidence of similar effectiveness in patients with HFpEF of drugs known to alleviate the severity of symptoms and reduce mortality and hospitalizations in patients with HFrEF. However, spironolactone (an aldosterone antagonist) has conflicting results. A meta-analysis of three randomized trials (TOPCAT, HOMAGE, Aldo-DHF) showed that spironolactone induced functional as well as structural improvement in patients with HFpEF [211], but in HFpEF, compared with HFrEF patients, spironolactone failed to show an overall benefit in reducing all-cause mortality but only a reduction in HF hospitalization rates [212]. These clinical data support the idea that HFpEF is not an incipient form of HFrEF [213] (Table 1).

A hope for improving patients’ state with HFpEF was represented by valsartan/sacubitril (angiotensin receptor—neprilysin inhibitors, ARNI), through the beneficial results shown in patients with HFrEF (Table 1). However, the PARAGON-HF study failed to demonstrate, in patients with HFpEF, a significant reduction in the risk of death from cardiovascular causes or in the hospitalization rate for HF decompensation [204]. In subgroup analysis, there was however a clinical benefit observed in patients with LVEF < 57% and in women [214]. Another study addressed valsartan/sacubitril, which included patients with HF with preserved or slightly impaired ejection fraction, treated for an episode of cardiac decompensation, PARAGLIDE-HF, which showed that this therapy is effective in reducing the level of natriuretic peptides in these types of patients, with a reduction in the number of cardiac decompensations, but it did not have statistical power to be able to provide data regarding the influence on mortality [215].

Nochioka et al. show that there is some evidence that statin (lipid-lowering medications) administration may reduce mortality in patients with HFpEF [216].

Chlorthalidone, a thiazide diuretic used in the treatment of hypertension, has been shown to reduce the occurrence of HFpEF in hypertensive patients [217].

Tafamidis, a drug developed for the treatment of transthyretin amyloid cardiomyopathy (ATTR-CM), showed according to the ATTR-ACT study that if it is used from the early stage of the disease, it reduces mortality and the number of hospitalizations, these benefits being due to the reduction in heart contractile dysfunction [218].

In HFpEF, despite global preserved LVEF, hypocontractile and hypercontractile subtypes have been described. Myosin modulators are being developed for these HFpEF phenotypes. They either activate (omecamtiv mecarbil) or inhibit (mavacamten) cardiac contractility by binding to β-cardiac myosin [163,219,220,221,222].

Soluble guanylate cyclase (sGC) stimulators, effective vasodilatory substances, have been successfully used in the treatment of primary pulmonary arterial hypertension [223,224]. One of the representatives, vericiguat, was studied in the therapy of patients with HFpEF, considering the mode of action similar to that of NO. There were two studies (SOCRATE-Preserved and VITALITY-HFpEF), but these did not demonstrate any benefit. However, when enrolling patients, these studies did not differentiate between patients without or with PH [225,226].

The real progress in the therapy of these patients was achieved with the appearance of sodium–glucose co-transporter 2 (SGLT2) inhibitors (iSGLT2) that act at the level of the proximal renal tubules, inhibiting the reabsorption of glucose from the primary urinary and promoting natriuresis (Table 1). These molecules, developed for the treatment of diabetes mellitus, have proven positive effects on the pathogenetic changes in HF. The mechanisms by which it improves the evolution of patients with heart failure along the entire spectrum, from HFrEF to HFpEF, are complex and aim at hemodynamic and metabolic aspects. One of the first mechanisms described was the reduction in blood pressure by approximately 4/1.6 mmHg, this reduction not being accompanied by a compensatory increase in heart rate, which leads to the speculation that the activation of the sympathetic nervous system does not occur [227]. The reduction in blood pressure was attributed to the reduction in circulating volume and plasma sodium concentration due to the effects of iSGLT2 at the renal level [228]. However, this reduction in blood pressure is not sufficient to explain the rapid favorable evolution of patients with HF, given that some of them were normotensive; moreover, clinical studies have shown that the reduction in blood pressure is even more modest than 1 mmHg for both systolic and diastolic blood pressures [229]. However, the reduction in volume, obtained by increasing diuresis, can be an explanation, the reduction in plasma volume being approximately 7.3% [230]. Simultaneously with the reduction in the circulating volume, a reduction in the interstitial fluid volume was also observed, a reduction more pronounced than that obtained with diuretics [230]. Another consequence of iSGLT2 administration is the reduction in arterial stiffness, an important component in the pathogenesis of HF, mainly by improving endothelial function [231]. Beyond the hemodynamic effects, there are also direct effects at the level of myocytes. Reducing the sodium and calcium loading of myocytes by reducing the expression of NEF1 and NEF3 (sodium–hydrogen exchangers) decreases hypertrophy and the risk of ischemia [232]. The formation of ketones as a result of the use of iSGLT2 favors the use of beta-hydroxybutyrate as an energy substrate by the myocyte to the detriment of fatty acids, a fact that optimizes cardiac oxygen consumption [233]. In addition, the reduction of inflammation was observed by monitoring the levels of C-reactive protein (CRP), TNF-α, and IL-6 [234]. Consequently, as a result of these mechanisms, the reduction in LV mass, reduction in parietal fibrosis, and improvement of diastolic function and parietal stress were observed [235,236].

Two representatives of this class have been studied in HF, dapagliflozin, and empagliflozin. They have been proven to reduce the risk of both cardiovascular death and worsening episodes of heart failure in patients with HFrEF [237]. After the success they had in the treatment of patients with HFrEF, iSGLT2 was also studied in patients with LVEF > 40%. The DELIVER study demonstrated that dapagliflozin reduced the combined risk of worsening heart failure or cardiovascular death in patients with HFmrEF or HFpEF [238]. The same positive results were obtained in the EMPEROR-Preserved study, which also enrolled patients with HF and LVEF > 40%. The authors conclude that empagliflozin reduces these risks in patients with HFmrEF and HFpEF regardless of the presence of diabetes mellitus [239]. Later, these studies had sub-analyses on the group of patients with LVEF > 50%, the results confirming the same favorable effects regardless of LVEF [240]. We have to wait for the results of future studies carried out with the declared objective of measuring the effects strictly on patients with HFpEF. Currently, the conclusion of these meta-analyses was sufficient to recommend iSGLT2 in the treatment of patients with HFpEF. Both ESC and AHA/ACC/HFSA guidelines now support iSGLT2 treatment in patients with HFpEF, the recommendation being a strong one for the ESC guideline [241] and a moderate one for the AHA/ACC/HFSA guideline [23]. The favorable effects are due to the reduction in myocardial hypertrophy and fibrosis, the improvement of diastolic dysfunction and LV filling, and the improvement of myocyte metabolism [242].

The lack of effective medication has led to an extensive search for new effective molecules, with preliminary positive data for antioxidants, antifibrotic drugs, anti-inflammatory drugs, microARNases, PAI-1 inhibitor, endothelin receptor A and B antagonists, and mitochondrial-targeted drugs [243] (Table 2). Although the results are promising, further investigations are needed.

New possible therapeutic pathways have emerged with the exploration of senolytic therapeutic agents. A senolytic molecule can selectively induce senescent cell apoptosis and thereby improve human health [244]. There are molecules in different experimental phases that promise favorable effects in this direction. As previously mentioned, senescence plays an important role in the occurrence of HFpEF. It affects the arteries both at the endothelial level, where it is responsible for the development/progression of endothelial dysfunction, and at the level of smooth muscle cells, promoting arterial stiffness and the development of atherosclerosis [245]. Cellular senescence also affects the heart by altering myocytes (hypertrophy and decreased contractility) and fibroblasts (increased fibrosis) [246].

One of these molecules is ABT-263, also known as Navitoclax. Administration of Navitoclax to rats with signs of premature aging had systemic effects manifested by decreased systemic inflammation and circulating senescent cells. In addition, it led to the attenuation of myocardial remodeling, especially of endothelial dysfunction and fibrosis, and to the reduction of BNP levels [247]. There are other senolytic molecules with favorable cardiovascular effects: digoxin [248], alvespimycin or 17-dimethylaminoethylamino-17-demethoxygeldanamycin [249], vaccination against glycoprotein nonmetastatic melanoma protein B [250]. Although targeting cellular senescence is a new field, the development of therapeutic agents can improve the prognosis and quality of life of patients with HFpEF, even beyond the effects on the cardiovascular system.

### 14.2. Non-Pharmacological Therapy

The non-pharmacological methods are similar to those used in HFrEF and aim to correct the risk factors for HF. They aim at a healthy, Mediterranean-type diet, with a low intake of salt and a caloric intake that allows optimizing body weight. Along with the diet, regular physical exercises (150 min/week) and quitting smoking are absolute recommendations [1].

Patients with HF frequently present associated cardiac pathologies, among them are arrhythmias. Regardless of the type of HF, atrial fibrillation is the most frequently encountered, contributing to additional hemodynamic deterioration and decreased exercise capacity. Antiarrhythmic medication often has limited effects. To maintain the sinus rhythm, different ablation techniques have been tried, among them the isolation of the pulmonary veins with positive results [251]. Interatrial shunt procedures and devices are also therapeutic options for the LA myopathy phenotype of HFpEF [163,222]. Thus, the transcatheter transvenous placement of an interatrial shunt device (IASD) can improve hemodynamics during exercise, thus leading to improved functional capacity and a better quality of life [252].

For ischemic etiology, in apatients with angina, despite optimal medical treatment, myocardial revascularization is recommended [180].

The complex pathophysiology of HFpEF requires the development of multiple treatment strategies aimed at the specific mechanisms of the disease, and the treatment of comorbidities having a key role in these patients.

## 15. Challenges, Findings and Limitations

The main challenges were to compile very large amounts of information, to write in a uniform way, and to accommodate the different interpretations regarding HFpEF. Another challenge was related to the fact that clinical trials do not classify patients with HFpEF into clinical phenotypes, so medications that apparently had no effect could actually be effective on certain phenotypes.

The main findings of this study were the extensive description of clinical and biological phenotypes and how the use of artificial intelligence can help to characterize them more precisely in the future, along with a review of existing therapeutic resources and possible ways of developing them in the near future.

One of the main limitations is related to the fact that the treatment of patients with HFpEF, according to the guidelines, also includes the treatment of comorbidities, a topic that was not addressed in this review. Another limitation is related to the definition of HFpEF. The different interpretations of the diagnostic elements of HFpEF, in different studies and clinical trials, make the comparative analysis of the results difficult. It is possible that our search strategy failed to retrieve relevant studies, because literature of interest in languages other than English were not included.

## 16. Future Perspective and Conclusions

HFpEF is a systemic syndrome with a poor prognosis. The number of patients with HFpEF has been on a constant increase in the last decades; at the present moment, half of the newly diagnosed patients with HF have the phenotype with preserved ejection fraction. There is a reasonable belief that HFpEF will become the dominant form of HF. With the increase in morbidity and mortality related to HFpEF, and the costs borne by society have increased proportionally. This is also due to the fact that, until now, there has been no effective treatment, with physicians having only symptomatic therapies available. That is why the primary intervention to identify modifiable risk factors, for the prevention of the disease, as well as understanding the heterogeneity and phenotypic classification of patients with HFpEF, with the aim of rapid recognition and initiation of an early therapy of these patients, becomes important. “Classical” drug therapies have limited effects, but innovative therapies (iSGLT2) offer hope due to already proven positive results. Thus, at the moment, iSGLT2 together with new therapeutic perspectives (senolytic therapies) represents hope for improving the evolution of patients with HFpEF. Therefore, there is no universal approach for patients with HFpEF, the ideal approach being an individualized therapy, adapted to their phenotype. In conclusion, solving the problem of HFpEF means a satisfactory definition, the use of all means, including artificial intelligence for the extensive characterization of clinical and biological phenotypes and the detection of specific HfpEF ECG, ultrasound patterns, along with the development of new classes of drugs addressed to specific pathological pathways.

## Figures and Tables

**Table 1:** Treatment recommendations for patients with HFpEF. ACC/AHA—American College of Cardiology/American Heart Association; ARNi—angiotensin receptor—neprilysin inhibitors; ESC—European Society of Cardiology; HF—heart failure; iSGLT2—sodium–glucose co-transporter 2 inhibitors; LVEF—left ventricular ejection fraction; MRA—mineralocorticoid receptor antagonists; NA—not applicable. green color—is recommended or is indicated; yellow color—should be considered; orange color—may be considered; grey color—not applicable.

Drug Class	ESC Indication	ACC/AHA Indication	Purpose	Observations
**Diuretics**	I	1	alleviate symptoms	fluid retention
**iSGLT2**	I	2a	decreasing HF hospitalizations and cardiovascular mortality	
**MRAs**	NA	2b	decrease hospitalizations	patients with LVEF on the lower end of this spectrum
**ARNi**	NA	2b	decrease hospitalizations	patients with LVEF on the lower end of this spectrum

**Table 2:** Possible future therapies for patients with HFpEF. LV—left ventricle; PAI-1—plasminogen activator inhibitor-1; TGF-β—transforming growth factor beta.

Possible Future Therapies			
Type	Mechanism	Effect	References
Myosin modulators	binding to β-cardiac myosin	modulate cardiac contractility	[163,219,220,221,222]
Soluble guanylate cyclase stimulators	guanylate cyclase stimulator	vasodilator effect	[223,224]
Senolytic therapy	induce senescent cell apoptosis	improves cardiac function	[244,245,246,247,248,249,250]
PAI-1 inhibitor	TGF-β and plasminogen-mediated pathways	reduces cardiac fibrosis	[243]
Endothelin receptor A and B antagonists	improves hemodynamics	cardiac remodeling	[243]
Mitochondrial-targeted drugs	increases mitochondrial energy	improvement of LV systolic function	[243]
